# Herbivore‐mediated negative frequency‐dependent selection underlies a trichome dimorphism in nature

**DOI:** 10.1002/evl3.157

**Published:** 2020-01-09

**Authors:** Jay K. Goldberg, Curtis M. Lively, Sonya R. Sternlieb, Genevieve Pintel, J. Daniel Hare, Michael B. Morrissey, Lynda F. Delph

**Affiliations:** ^1^ Department of Biology Indiana University Bloomington Indiana 47405; ^2^ Wesleyan University Middletown Connecticut 06459; ^3^ University of Guelph Guelph Ontario N1G2W1; ^4^ Department of Entomology University of California Riverside California 92521; ^5^ School of Biology University of St. Andrews St. Andrews KY16 9AJ UK

**Keywords:** balanced polymorphism, *Datura wrightii*, glandular trichomes, plant–herbivore interactions

## Abstract

Negative frequency‐dependent selection (NFDS) has been shown to maintain polymorphism in a diverse array of traits. The action of NFDS has been confirmed through modeling, experimental approaches, and genetic analyses. In this study, we investigated NFDS in the wild using morph‐frequency changes spanning a 20‐year period from over 30 dimorphic populations of *Datura wrightii*. In these populations, plants either possess glandular (sticky) or non‐glandular (velvety) trichomes, and the ratio of these morphs varies substantially among populations. Our method provided evidence that NFDS, rather than drift or migration, is the primary force maintaining this dimorphism. Most populations that were initially dimorphic remained dimorphic, and the overall mean and variance in morph frequency did not change over time. Furthermore, morph‐frequency differences were not related to geographic distances. Together, these results indicate that neither directional selection, drift, or migration played a substantial role in determining morph frequencies. However, as predicted by negative frequency‐dependent selection, we found that the rare morph tended to increase in frequency, leading to a negative relationship between the change in the frequency of the sticky morph and its initial frequency. In addition, we found that morph‐frequency change over time was significantly correlated with the damage inflicted by two herbivores: *Lema daturaphila* and *Tupiochoris notatus*. The latter is a specialist on the sticky morph and damage by this herbivore was greatest when the sticky morph was common. The reverse was true for *L. daturaphila*, such that damage increased with the frequency of the velvety morph. These findings suggest that these herbivores contribute to balancing selection on the observed trichome dimorphism.

**Impact Summary**

We present a long‐term observational study of morph frequency changes in naturally dimorphic populations of *Datura wrightii*. We were able to determine that negative frequency‐dependent selection—rather than drift, migration, or directional selection—is the main contributor to the maintenance of this dimorphism over the past 20 years. We also sampled herbivory across our sample of populations and found evidence suggesting that the damage inflicted by two species of specialist herbivores may underlie this selective regime.

Negative frequency‐dependent selection (NFDS)—a selective regime in which rare morphs are favored over common ones—has been implicated in the maintenance of many polymorphic traits observed in nature (Delph and Kelly 2014; Brisson 2018). Under NFDS, morph frequencies are expected to fluctuate as a consequence of fitness being conditional on frequency, with rare morphs having higher fitness relative to common morphs. The outcome is a balanced polymorphism. In plants, floral‐color polymorphisms can be derived from pollinator‐mediated NFDS (Gigord et al. 2001). Similarly, parasites have been shown to generate NFDS among genetic variants in a clonal snail species (Koskella and Lively 2009). NFDS mediated by societal conflict has even been suggested to play a role in determining the frequency of left‐handed humans within countries (Faurie and Raymond 2005).

Studies of NFDS have used an array of experimental, genetic, and modeling approaches to examine the ways in which this form of selection can favor polymorphism (Kazancioğlu and Arnqvist 2013; Lindtke et al. 2017; Sato et al. 2017; Sato and Kudoh 2017). Experimental manipulations of morph frequencies and subsequent fitness observations in natural populations of organisms provide some of the strongest evidence that NFDS governs some stable polymorphisms. Population‐genetic studies allow for the detection of balancing selection in natural populations but cannot determine if NFDS is the underlying mechanism (Tian et al. 2002). In this paper, we use long‐term measurements of phenotypic variation in natural populations to determine whether a plant‐defense dimorphism is governed by NFDS. We likewise investigate whether the observed changes over time are correlated with environmental factors—both abiotic and biotic.

Plant defenses are diverse, with most plant species using chemicals, physical structures (e.g., thorns), or indirect strategies such as the attraction of the natural enemies of herbivores (Chen 2008; Aljbory and Chen 2018). These defenses can vary both within and between plant populations (Rios et al. 2008), making them an ideal system for studying the selective regimes that generate and maintain balanced polymorphisms. In fact, NFDS has already been shown to maintain diversity in plant defenses against both herbivores and pathogens (Siemens and Roy 2005; Sato and Kuboh 2017).

Our study species, *Datura wrightii*, has dimorphic trichomes (glandular vs. non‐glandular) and individual phenotypes are easily observed in the field. Because the morph frequencies of over 50 populations had already been measured and published (Hare and Elle 2001), we were able to examine how morph frequencies changed over a nearly 20‐year period within a large geographic range, and to test whether this change was related to NFDS, drift, directional selection, or migration (see Table 1). NFDS would not necessarily increase the mean or the variance in morph frequencies over time, and we would expect a negative relationship between the change in frequency and initial frequency. Populations can vary substantially in their dynamics, fluctuating around different equilibrium frequencies, remaining stable over time, or being more or less governed by drift/migration based upon local conditions (population size, proximity to neighboring populations, etc.); thus in order to detect NFDS, we examined a large number of populations over a relatively long period of time. If morph frequencies in our sample of populations were governed by genetic drift, the mean frequency of the morphs need not change over time, but the among‐population variance in morph frequency would increase as different populations move toward random fixation of one, or the other, of the two morphs. Directional selection should lead to an increase in the frequency of one of the morphs in the metapopulation, and a corresponding decrease in variance. Migration, on the other hand, should lead to a decrease in variance in morph frequency across the metapopulation.

**Table 1:** Predicted effects of different evolutionary forces on various aspects of morph frequencies and population structure. Δp refers to the change in morph frequency over time. Morph frequencies of *Datura wrightii* across California match the predictions of negative frequency‐dependent selection

	Δp	Δp versus *p*	*Variance in p*
Drift	No net change	Slope = 0	Increase
Migration	No net change	Negative slope	Decrease
Directional Selection (‐)	Negative	Positive slope	Decrease
Negative Frequency‐Dependent Selection	No net change	Negative slope	No change
**Field Observations**	**No net change**	**Negative slope**	**No change**

## Methods

### STUDY SYSTEM

*Datura wrightii* is a solanaceous shrub native to the desert regions of the United States and Northern Mexico. While adult plants usually possess non‐glandular trichomes and are velvety to the touch, a morph with glandular trichomes is present in populations across California, although the glandular morph is notably absent from populations in the eastern part of *D. wrightii*’s range (Hare and Elle 2001; JK Goldberg, pers. obs.). Hare and Elle (2001) surveyed the frequency of the two morphs in many populations from 1997 to 1999. In eight of these populations, they also investigated net reproductive rate of the two morphs and whether there was a change in morph frequency between 1998 and 2002 (Hare and Elle 2004). They found that high frequencies of sticky plants (up to 89%) could persist despite higher seed production by velvety plants. Because the higher reproductive rate of velvety plants did not correspond with a decrease in sticky frequency, they concluded that 5 years was insufficient time “to fully understand the dynamics of the trichome dimorphism in *D. wrightii*.”

Glandular trichomes have a strong impact on the herbivores of *D. wrightii*. Glandular trichomes confer resistance to flea beetles (*Epitrix* sp.) but increase susceptibility to mirid suckflies (*Tupiochoris notatus*; Hare and Elle 2002). They do not influence a specialist beetle (*Lema daturaphila*; Hare and Elle 2002) but do slow the growth of hornworms (*Manduca sexta*; van Dam and Hare 1999). The sticky‐velvety dimorphism exhibits single locus Mendelian inheritance, with the dominant allele encoding the sticky (glandular) phenotype and the recessive allele producing the velvety (non‐glandular) phenotype (van Dam et al. 1999). The phenotype also changes over ontogeny; all plants produce glandular trichomes as seedlings and homozygous recessive genotypes transition to their adult velvety phenotype thereafter (van Dam et al. 1999).

### FIELDWORK AND DATA COLLECTION

**Figure 1:**
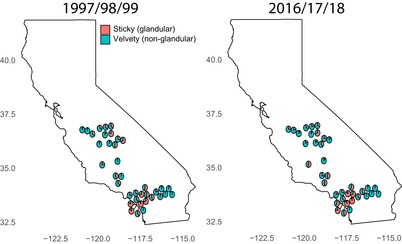
Maps showing the morph frequency of populations of *D. wrightii* visited in 2016, 2017, and 2018. (A) The average frequencies measured by Hare and Elle (2001) in 1997–1999 and (B) the average frequency measured in 2016–2018. The location of each population is shifted slightly to avoid overlapping pies.

We visited a total of 37 *D. wrightii* populations originally studied by Hare and Elle (2001) in 1997–1999 (shown in Fig. 1 and Supporting Information Fig. S1), in July/August of 2016 (*N* = 34; Fig. 1A), 2017 (*N* = 32; Fig. 1B), and 2018 (*N* = 34; Fig. 1C). We determined the trichome phenotype of all plants within each population via sight and touch. We chose to conduct whole‐population censuses in order to minimize effects of sampling error on our analysis, as has been done in prior studies on density‐dependent effects (Freckleton et al. 2006). For populations that were visited in multiple years (*N* = 34), we used the average sticky frequency across years in our analysis. Otherwise, we used the observed frequency for a single year (2 in 2016; 1 in 2018).

At nine populations in 2016, we also surveyed the arthropods present on a single branch (encompassing inner, outer, top, and bottom sections of each plant) of a sample of 48 plants in each population. For populations with fewer than 48 plants, arthropod surveys were conducted on all plants. In 2017, we conducted arthropod counts at 22 populations (shown in Supporting Information Fig. S1), reduced our sample of plants to 36 per population, and estimated the percent of leaf area that had been damaged by *Epitrix*, *L. daturaphila*, *M. sexta*, and *T. notatus* on our sample plants using published descriptions of these species’ damage (Elle and Hare 2000). Arthropod counts and damage estimates were then both averaged within each population for use in statistical analyses. The density of these herbivore populations varies between populations and across the growing season (Elle and Hare 2000). The timing of our measurements was during the time of year when multiple herbivore species are typically present in multiple habitats, although it did not coincide with peak activity of any species (Elle and Hare 2000). Environmental variables (temperature and precipitation) for our study populations were obtained from the PRISM database (Oregon State University, Corvallis, OR, USA). All modeling and statistical analyses were conducted in R.

### STATISTICAL ANALYSIS: FREQUENCY DEPENDENCE, MIGRATION, AND DRIFT

To screen for the presence of NFDS, we compared the change in frequency (Δp) and the initial frequency (*p*) for each population. We used Pearson's correlation to determine if the Δp of a population could be predicted by its *p*. A significant negative correlation between these two values is predicted if NFDS is acting upon these populations. Although this relationship can be indicative of NFDS, it could also be produced by strong migration in the presence of heterogeneous selective pressures. However, unlike NFDS, strong migration should act to reduce variation in morph frequency between populations (Maruyama 1970, 1972). Thus, we used Bartlett's test to compare the variance in morph frequencies between our two time points. We also used Mantel tests to test for the presence of a correlation between geographic distance and morph‐frequency difference, a method commonly used in population‐genetic studies (Diniz‐Filho et al. 2013). To check for the presence of directional selection, we used a paired sample *t*‐test to look for a change in the mean sticky frequency over time. If the mean increased it would indicate positive selection for the sticky morph, while if it decreased it would indicate negative selection.

If drift were the dominant force, we expected to see a significant increase in the variance of morph frequencies driven by random fixation of one morph or the other in various populations. We simulated drift by back calculating allele frequencies from the observed morph frequencies (defined as a value of 0 or 1 given to each "individual") for sets of populations that matched our observed data (37 populations with an average starting size of 62 individuals). We ran 10,000 iterations of five generations and calculated the mean number of extinction/fixation events across all iterations. Each “generation” was the creation of a new morph frequency set using that population's morph frequency in the previous generation to randomly generate the allele frequency in the next generation. This method was designed to emulate the tracking of alleles. *Datura wrightii* individuals live for 3–5 years (Hare and Elle 2004), thus five generations is a reasonable estimate for what occurred in the nearly 20‐year period of our study. We then calculated the percentile in which our observed number of fixations and change in the variance fell within the simulation's output.

### STATISTICAL ANALYSIS: EFFECT OF UNCERTAINTY

Although we determined the trichome‐morph phenotype of all plants at each population we visited, there is still room for uncertainty in our morph‐frequency estimates because of the presence of a dormant seed bank of unknown size at each population. The error effects generated by this uncertainty can generate a negative relationship between starting frequency and the change in frequency (Freckleton et al. 2006). To address if our results could have been generated by error effects alone, we produced a simulation of morph‐frequency changes in the absence of drift and selection. This simulation randomly generated allele frequencies for a recessive morph (p) in 37 populations (matching our sample size) with 62 adult plants each (the average starting size in Hare and Elle 2001) using a uniform distribution. A uniform distribution was used given that the actual distribution of allele frequencies was unknown. We then calculated morph frequencies (*p* ^2^) for this set of populations and repeated this process to generate a second “timepoint.” Next, we calculated the slope of the relationship between the starting morph frequency and the change in morph frequency for 10,000 iterations of this simulation. We then compared these slopes to the slope we observed in our field data.

### STATISTICAL ANALYSIS: EFFECT OF ENVIRONMENT

Environmental variables were used as independent factors in linear regressions with population size, current sticky morph frequency, or change in sticky frequency as the dependent variables. To analyze the effect of herbivores, the average number of herbivores per sampled plant was calculated for each population and used as the independent variable with the dependent variables listed previously. Each species of herbivore (*T. notatus*, *M. sexta*, *L. daturaphila*) was analyzed separately. In cases where data were not normally distributed, a Spearman's ranked correlation was used.

## Results

### DRIFT, FREQUENCY DEPENDENCE, AND MIGRATION

**Figure 2:**
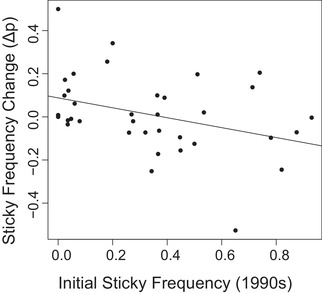
The negative relationship between changes in sticky morph frequency from the late 1990s to present (2016–2017) and the initial sticky frequency measured by Hare and Elle (2002).

The trichome dimorphism was retained over the 20‐year period between the late 1990s and 2018, with 34 out of 37 populations being dimorphic in 2018. The frequency of sticky plants observed in 2016–2018 differed from the frequency seen in the late 1990s (those observed by Hare and Elle 2001; Fig. 1). The change in the sticky frequency was found to be negatively correlated with the initial sticky frequency (Fig. 2), which is consistent with the operation of NFDS (β1 = −0.230, *R* ^2^ = 0.132, *p* = 0.027). Two populations that were dimorphic in the 1990s became monomorphic (a different morph became fixed in each) during the study period, but these changes were concurrent with extreme population bottlenecks (Lemoore dropped from 53 individuals to 12, and Coyote Pass in Lake Perris SRA from 192 to 3 in 2018) indicating that these fixation/extinction events are likely caused by drift rather than directional selection (Ellstrand and Elam 1993). One population remained entirely composed of velvety plants the whole time, indicating that directional selection may be the dominant force in that location (Wilson Canyon Wash in Joshua Tree National Park). However, it is also possible that the sticky morph has never been introduced into this area. Our simulation of drift generated an expected number of extinction/fixation events equal to 6.5; although the number of fixations we observed (*N* = 2) was within the range generated by the simulation, only 0.1% of iterations generated 2 or fewer fixation events. We also used our simulation to calculate the expected positive change in variance after five generations, which was 0.006. In contrast, in our field data we observed a small decrease in variance (change = –0.005). While this value was within the range of values produced by our simulation, it was well within the lower tail, with only 6% of values being less than our observed value. Together, these results demonstrate that while drift is capable of influencing *D. wrightii* morph frequency, it is likely not the dominant force maintaining the dimorphism across California. The observed changes in morph frequencies could also be generated by heterogeneous directional selection in the presence of substantial migration or even drift alone; hence, we also conducted analyses to determine whether migration was acting to reduce variance in the sticky frequencies of our populations. Bartlett's test found that the difference in the variance across these time periods was not significant (*K* ^2^_1,72_ = 0.034, *p* = 0.854) and a paired *T*‐test also demonstrated that means did not differ between the time periods (*t* = −0.444, *p* = 0.660; Fig. S2). These results indicate that neither migration nor directional selection is the dominant force in the metapopulation. Mantel's correlations showed that in neither the 1990s or 2010s was there a relationship between geographic distance and morph‐frequency similarity (1990s: *r* = −0.027, *p* = 0.704; 2010s: *r* = 0.020, *p* = 0.289), indicating that populations that are geographically close are not more similar with respect to their morph frequencies than distant populations. As such, our results match our expectations for NFDS being the dominant force governing the California trichome dimorphism (Table 1).

### EFFECT OF UNCERTAINTY

Our simulation generated an average slope of −0.023. This is consistent with the expectation that error‐effects alone can generate a negative slope; however, the minimum slope generated by our simulation was −0.199, a value less steep than our observed slope of −0.230. This demonstrates that uncertainty‐driven error effects are likely not sufficient to generate the relationship we observed in natural populations of *D. wrightii*.

### CORRELATIONS WITH ENVIRONMENT AND HERBIVORES

**Figure 3:**
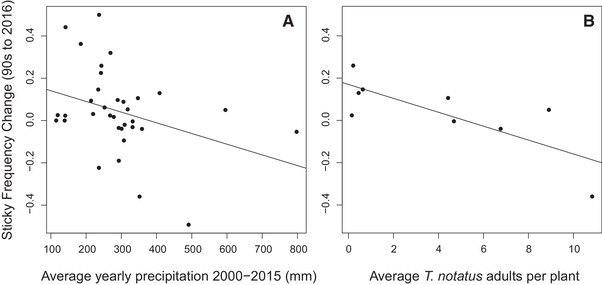
Both the (A) long‐term average yearly precipitation and (B) average number of mirid adults per plant were found to be negatively correlated with the change in the sticky morph frequency from Hare and Elle's (2001) observations in the late 1990's and our own.

In 2016, we found that the average yearly precipitation for the 16‐year period between Hare and Elle's (2001) study and our own (2000–2015) was negatively correlated with the change in the sticky‐morph frequency during that time (Spearman's rho = −0.392, *p* = 0.022, *df* = 32; Fig. 3A), indicating that the frequency of the sticky morph increased in drier regions. The average maximum (*R* ^2^ = 0.270, *p* = 0.122, *df* = 32), minimum (*R* ^2^ = 0.046, *p* = 0.794, *df* = 32), and mean temperatures (*R* ^2^ = 0.222, *p* = 0.207, *df* = 32) during this period were not correlated with observed morph‐frequency changes. Only the average number of *T. notatus* present on plants was found to be negatively correlated with changes in the sticky morph frequency in 2016 (*R* ^2^ = −0.772, *p* = 0.015, *df* = 7, Fig 3B). Numbers of *M. sexta* and *L. daturaphila* were not related to morph‐frequency changes (*M. sexta*: *R* ^2^ = −0.338, *p* = 0.373, *df* = 7; *L. daturaphila*: Spearman's rho = −0.45, *p* = 0.230). No herbivore or environmental variable was found to be correlated with the sticky frequencies observed in 2016 (Supporting Information Table S1).

**Figure 4:**
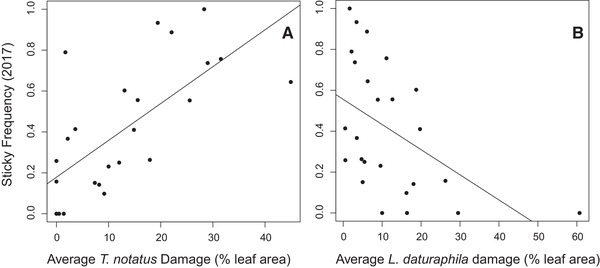
The sticky frequencies measured in 2017 were correlated positively with the average damage from (A) *Tupiochoris notatus* damage and negatively with that of (B) *Lema daturaphila*.

In 2017 we found that *T. notatus* population counts were positively correlated with the sticky frequencies observed that year (*R* ^2^ = 0.628, *p* = 0.001, *df* = 23). When we examined the effect of herbivore damage, we found that both *T. notatus* (*R* ^2^ = 0.688, *p* < 0.001; *df* = 23; Fig. 4A) and *L. daturaphila* damage (*R* ^2^ = −0.498, *p* = 0.011, *df* = 23; Fig. 4B) were correlated with the current sticky frequency, although in opposite directions. *Tupiochoris notatus* damage was positively correlated with the sticky frequencies observed in 2017, and *L. daturaphila* damage was negatively correlated. This indicates that populations in which sticky plants are common are more likely to become infested with *T. notatus*, whereas populations in which the velvety morph is common are more likely to be damaged by *L. daturaphila* beetles. Herbivore populations and damage did not correlate with the changes observed between the 1990s and 2017 (Supporting Information Table S2).

## Discussion

Our results are in concordance with NFDS expectations. We found that a trichome dimorphism of *D. wrightii* was retained over a 20‐year period in natural populations in California, with both sticky and velvety morphs co‐occurring in most of our study populations. The frequency of the morphs changed over the 20‐year period: the rare morph tended to increase in frequency, and the common morph to decrease in frequency. Previous research with this system has shown that sticky individuals consistently produced fewer seeds than velvety individuals, leading researchers to hypothesize that directional selection would eventually drive the velvety morph to fixation, as may be the case in monomorphic populations in the eastern parts of the plant's range (Hare and Elle 2004). Our results reveal that this is not the case and suggest that the fitness of the *D. wrightii* trichome morphs may be conditional upon local morph frequencies and environmental factors.

Although we found evidence for NFDS to be the primary force maintaining this dimorphism, it is not the sole evolutionary force that these plant populations experience. The fixation of morphs in two populations, both of which experienced substantial decreases in population size, indicates that drift acts in small populations, as is often shown by theoretical models (Ellstrand and Elam 1993). We also have evidence that NFDS is not universally present, as one population always remained monomorphic, suggesting that the lack of the gene for stickiness and/or directional selection favored the velvety morph in this location. This is unsurprising given that the sticky morph has not been observed in *D. wrightii* populations outside of California (Hare and Elle 2001; JK Goldberg, pers. obs.).

We also found evidence that insect herbivores may act as agents contributing to NFDS. These findings are consistent with the literature, which has found that herbivore‐mediated NFDS governs plant‐defense polymorphisms (Berenbaum and Zangerl 1998)—including variation in trichome type (Sato and Kudoh 2017). The observational nature of our study however, represents a novel departure from the literature norm, which usually treats various experimental approaches as the "gold standard" for studying traits under NFDS (Gigord et al. 2001). Our method can be applied to field observations of morph‐frequency changes over time from populations of virtually any organism. Furthermore, we have shown that these data can easily be compared to environmental variables to screen for the drivers underlying NFDS, although we recognize the need for experimental evidence to conclusively demonstrate how environmental factors (precipitation, herbivores, etc.) shape selection on polymorphic traits.

Strong convergence on an equilibrium morph frequency is usually considered a characteristic of NFDS and low inter‐population morph‐frequency variance has been used to identify polymorphisms potentially governed by NFDS in natural populations (Le Rouzic et al. 2014). However, it is possible for the equilibrium morph frequency to vary between populations when selective agents are variable (Gosden et al. 2011). Our method, while sufficient to screen for the presence of NFDS, is unable to determine the equilibrium dynamics of this selection. Given that the sticky‐morph frequency is stable at some populations, yet quite variable between populations and across time in other populations, we can conclude that the equilibrium frequency of this trait is likely determined by local environmental factors such as precipitation, temperature, or herbivore densities. Given that we observed two fixation events, drift may also be able to overtake the effects of selection when a population becomes sufficiently small. Without further studies investigating the effects of various factors on sticky and velvety morph fitness and the details of how these factors are dispersed, these dynamics remain unknown.

We also found that susceptibility to *L. daturaphila* and *T. notatus* was associated with the 2017 sticky morph frequency in opposite directions. The higher the sticky frequency of a population, the greater was its average damage from *T. notatus*. This is unsurprising as *T. notatus* is adapted specifically to life on sticky hosts (Wheeler and Krimmel 2015). *Tupiochoris notatus* populations in 2016 were also negatively associated with the sticky frequency changes since the late 1990s, indicating that sticky frequencies decreased the most where *T. notatus* populations are currently large. Furthermore, our data showed that the current sticky frequency was negatively associated with a population's average susceptibility to *L. daturaphila*, suggesting that this herbivore prefers populations with lower sticky frequencies, although they will clearly feed and reproduce on both morphs. This result is surprising, given that previous studies found that plants of both morphs in six populations were roughly equally infested with *L. daturaphila* throughout the growing season (Elle and Hare 2000). Furthermore, laboratory experiments found that they were not deterred by the noxious compounds present in the glandular exudate (Hare 2005). Nonetheless, our results suggest that *T. notatus* and *L. daturaphila* may contribute to NFDS on dimorphic *D. wrightii* in California.

Prior research found that consumption of leaf tissue by *M. sexta* larvae was slower in the presence of the glandular trichome exudate of the sticky plants (van Dam and Hare 1999). As such, we anticipated high sticky‐frequency populations to be more resistant to *M. sexta*; however, we found no relationship between *M. sexta* damage and *D. wrightii* trichome morph frequencies. We found that *M. sexta* was surprisingly uncommon in the populations we sampled—especially compared to *L. daturaphila* or *T. notatus*—thus it is possible that they were simply too rare for a significant relationship to be observed. Given that *M. sexta* larvae on sticky plants spend more time in the early instars, during which time they are susceptible to predation (Kessler and Baldwin 2001), it is also possible that indirect effects mediated by predatory arthropods may underlie glandular‐trichome mediated resistance to *M. sexta* and that these effects may be absent in years or locations with low densities of herbivore predators.

All *Datura* species possess non‐glandular trichomes and some species—much like *D. wrightii*—vary with respect to trichome type and density, and produce both glandular and non‐glandular trichomes (Hare and Elle 2001; Kariñho‐Betancourt et al. 2015). A phylogenetic study found a correlation between trichome density and other defensive traits (Kariñho‐Betancourt et al. 2015). Studies of trichome‐bearing plants species have associated selection on trichomes (including both density and type) with insect herbivory (Pullin and Gilbert 1989; Maluf et al. 2001). In the case of *D. stramonium*, higher non‐glandular trichome densities have been shown to improve resistance against *Epitrix* flea beetles, leading to directional selection in damaged populations (Valverde et al. 2001). Further studies with this species have shown that different herbivores select for or against different phenotypic traits, thus demonstrating that variation in plant defense is driven by herbivore‐distribution heterogeneity, which generates a "selection mosaic" across the landscape (Castillo et al. 2014). Castillo and colleagues focus on the strength of directional selection pressures across populations, while ignoring the effects of within‐population variation. Our results, showing that the frequency of glandular trichome‐bearing plants was correlated with damage from two herbivore species, builds upon prior findings by demonstrating that within‐population genetic variation can generate heterogeneous selective pressures on plant defensive traits. Furthermore, given the correlation between trichome density and other defensive traits (Kariñho‐Betancourt et al. 2015), we suggest that future studies should address the degree to which variation in chemical defenses can impact selection on trichomes.

In conclusion, we have shown that researchers can test for the presence of NFDS using direct observations of natural populations over time. Our study revealed that the predictions of NFDS—maintenance of the dimorphism and a rare‐morph advantage—were met across most of our 37 natural populations. We also found evidence suggesting that effects related to two herbivore species may underlie this selective regime. This demonstrates the utility of this method for future investigations of the evolutionary dynamics of polymorphic populations.

Associate Editor: S. Wright

## Supporting information


**Figure S1**. Maps showing the *D. wrightii* populations visited in 2016, 2017, and 2018.

**Figure S2**. Graphs visualizing analyses of the effect of migration.

**Table S1**. Correlations between environmental/herbivore variables and the sticky frequency observed in 2016.

**Table S2**. Correlations between various herbivory measurements and the sticky frequency change from the 1990s to 2017.
