# Supplementary figures and images for: Herbivore‐mediated negative frequency‐dependent selection underlies a trichome dimorphism in nature
Source: Evol Lett. 2020 Jan 9;4(1):83–90. doi: 10.1002/evl3.157 (PMC7006469; doi:10.1002/evl3.157)

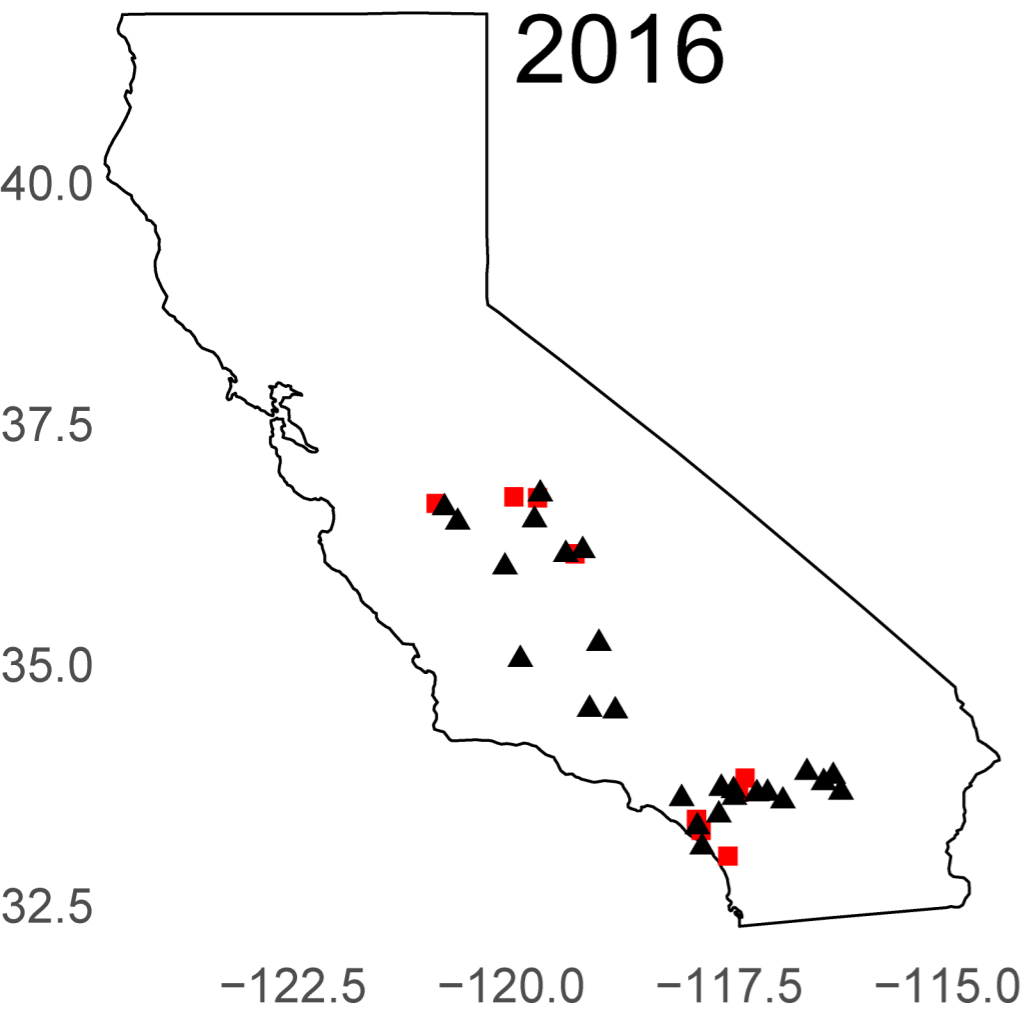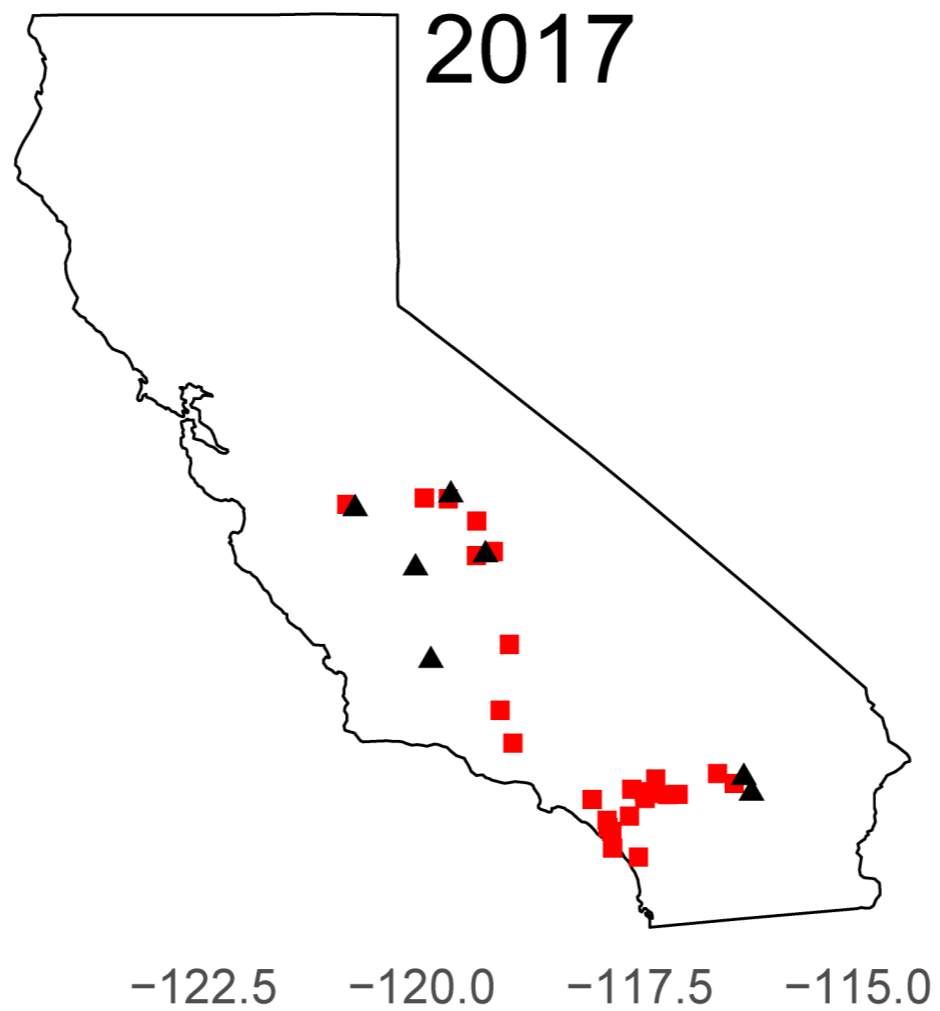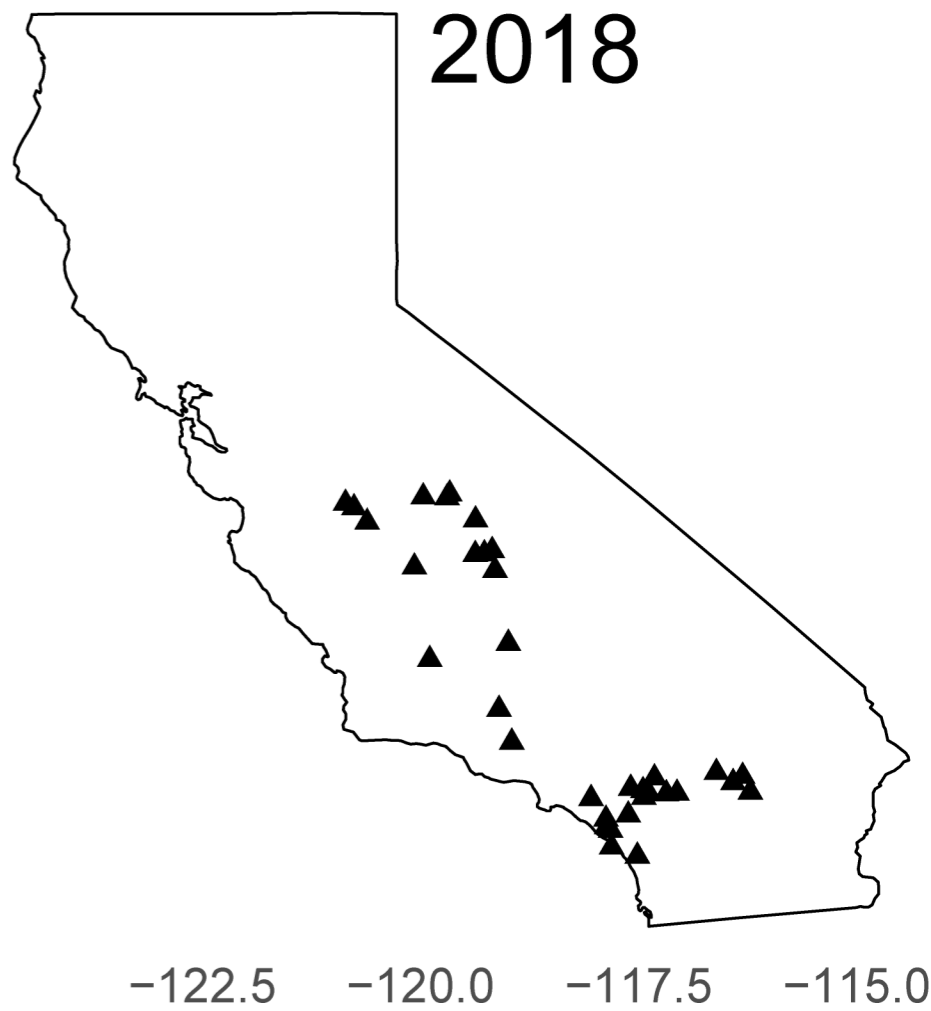

Supplement: Supplementary file 1 — Figure S1. Maps showing the D. wrightii populations visited in 2016, 2017, and 2018. [file EVL3-4-83-s001.pdf]

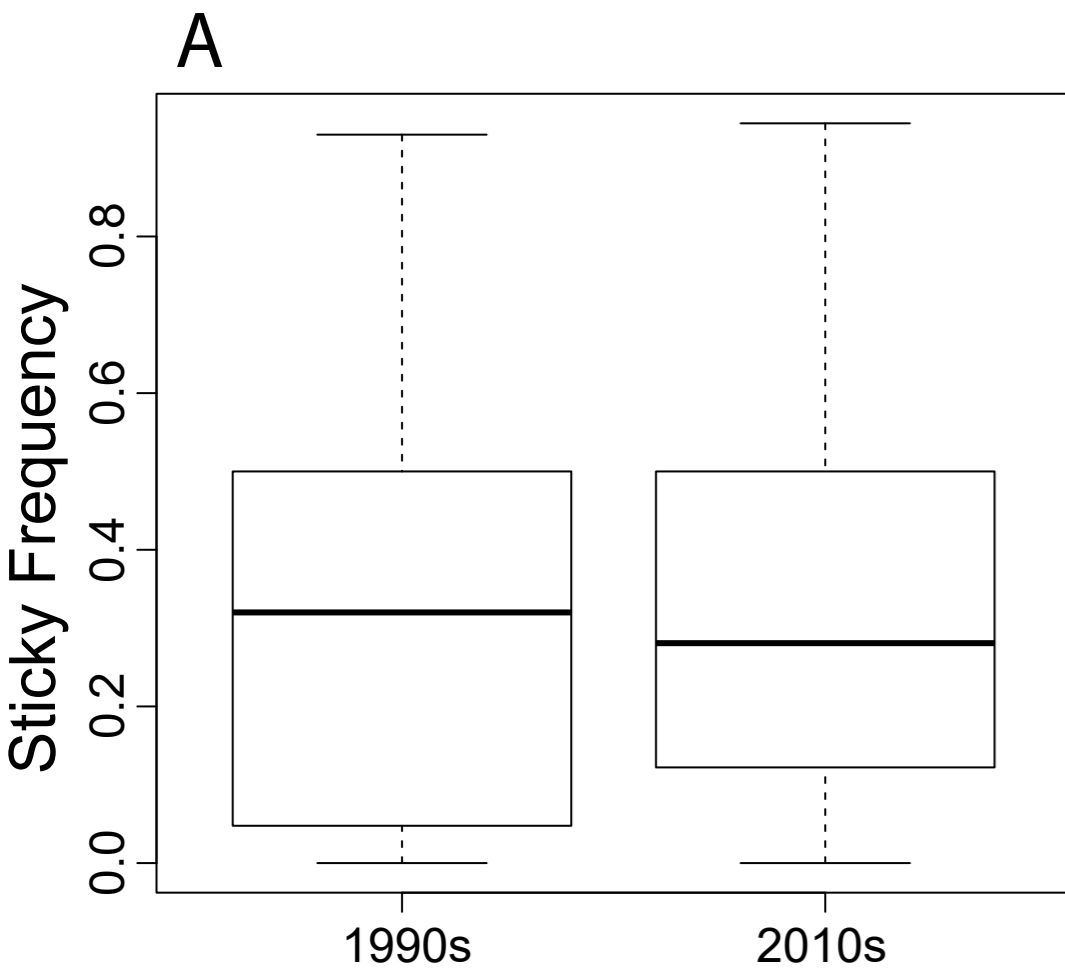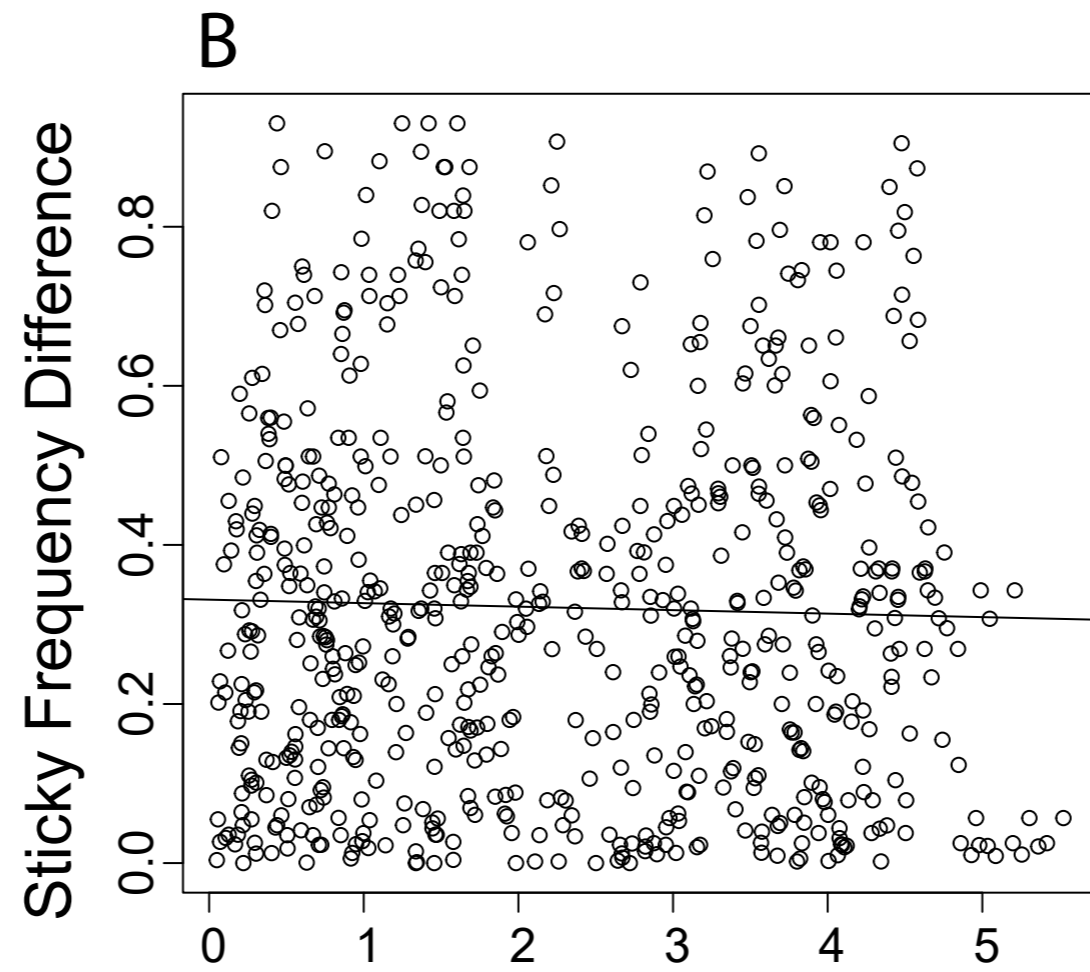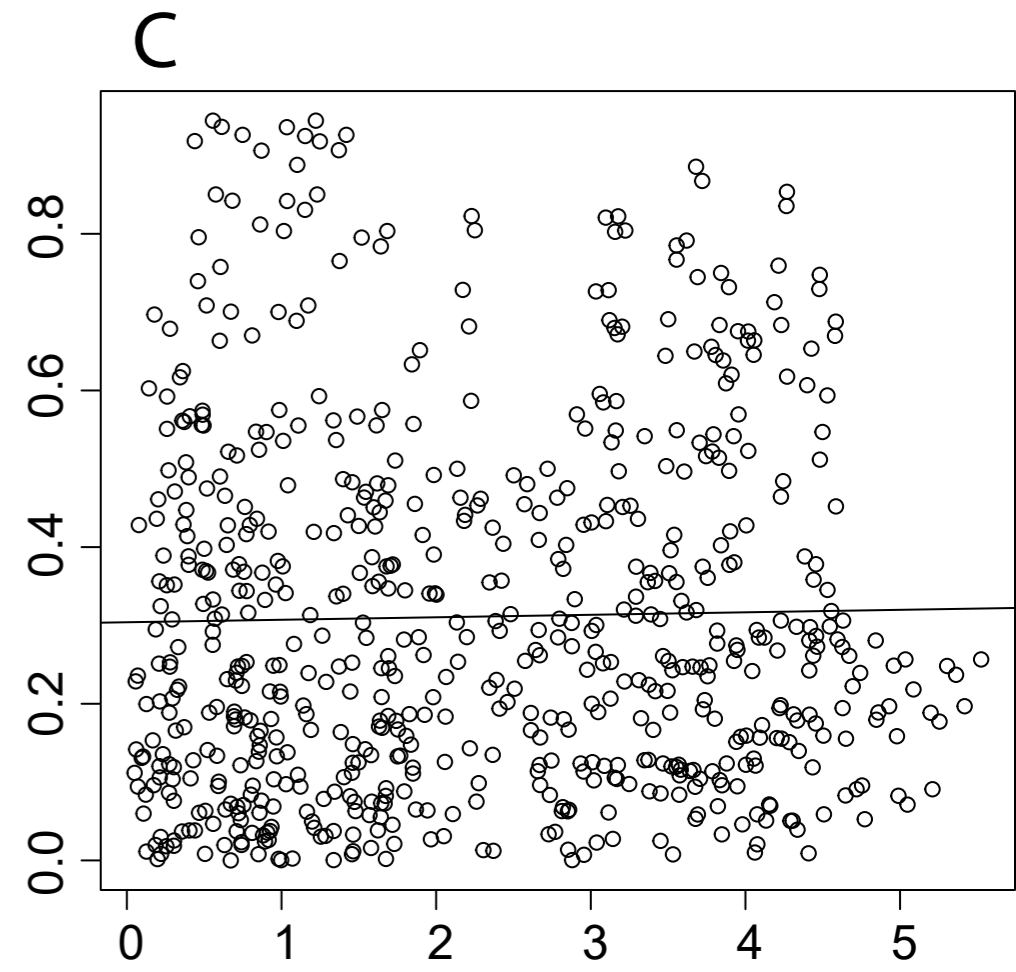

Geographic Distance (degrees)

Supplement: Supplementary file 2 — Figure S2. Graphs visualizing analyses of the effect of migration. [file EVL3-4-83-s002.pdf]
