# Supplementary material for: Herbivore‐mediated negative frequency‐dependent selection underlies a trichome dimorphism in nature
Source: Evol Lett. 2020 Jan 9;4(1):83–90. doi: 10.1002/evl3.157 (PMC7006469; doi:10.1002/evl3.157)
Supplement: Supplementary file 3 — Table S1. Correlations between environmental/herbivore variables and the sticky frequency observed in 2016. [file EVL3-4-83-s003.docx]

**Table S1.** Correlations between environmental/herbivore variables and the sticky frequency observed in 2016. None of these tests yielded a significant result.

| **Predictor Variable** | **Method** | **Test Statistic** | **p value** | **df** |
| --- | --- | --- | --- | --- |
| Average Yearly Precipitation (mm) | Spearman | 0.194 | 0.272 | 32 |
| Average Max Temp (C) | Pearson | 0.091 | 0.610 | 32 |
| Average Mean Temp (C) | Pearson | 0.049 | 0.783 | 32 |
| Average Min Temp (C) | Pearson | -0.045 | 0.801 | 32 |
| Manduca sexta larvae (count) | Pearson | 0.474 | 0.198 | 7 |
| Tupiochoris notatus adults (count) | Pearson | -0.008 | 0.985 | 7 |
| Lema daturaphila larvae and adults (count) | Spearman | -0.45 | 0.230 | 7 |
