# Supplementary material for: Herbivore‐mediated negative frequency‐dependent selection underlies a trichome dimorphism in nature
Source: Evol Lett. 2020 Jan 9;4(1):83–90. doi: 10.1002/evl3.157 (PMC7006469; doi:10.1002/evl3.157)
Supplement: Supplementary file 4 — Table S2. Correlations between various herbivory measurements and the sticky frequency change from the 1990s to 2017. [file EVL3-4-83-s004.docx]

**Table S2.** Correlations between various herbivory measurements and the sticky frequency change from the 1990s to 2017. None of these tests yielded a significant result.

| **Herbivore** | **Variable** | **Method** | | **Test Statistic** | | **p value** | | **df** | |
| --- | --- | --- | --- | --- | --- | --- | --- | --- | --- |
| Manduca sexta | larvae (count) | Pearson | -0.345 | | 0.092 | | 23 | |  |
|  | damage (log %) | Pearson | -0.174 | | 0.405 | | 23 | |  |
| Tupiochoris notatus | adult (count) | Pearson | 0.071 | | 0.735 | | 23 | |  |
|  | Damage (%) | Pearson | 0.104 | | 0.622 | | 23 | |  |
| Lema daturaphila | larvae + adults (count) | Pearson | -0.159 | | 0.449 | | 23 | |  |
|  | Damage (%) | Pearson | -0.251 | | 0.227 | | 23 | |  |
